# ITCH modulates SIRT6 and SREBP2 to influence lipid metabolism and atherosclerosis in ApoE null mice

**DOI:** 10.1038/srep09023

**Published:** 2015-03-17

**Authors:** R. Stöhr, M. Mavilio, A. Marino, V. Casagrande, B. Kappel, J. Möllmann, R. Menghini, G. Melino, M. Federici

**Affiliations:** 1 Department of Systems Medicine University of Rome “Tor Vergata”; 2 Medizinische Klinik I, University Hospital Aachen; 3 Department of Experimental Medicine and Surgery University of Rome “Tor Vergata”; 4 Medical Research Council, Toxicology Unit, Leicester LE1 9HN UK; 5 Center for Atherosclerosis, University Hospital “Policlinico Tor Vergata”, Rome

## Abstract

Atherosclerosis is a chronic inflammatory disease characterized by the infiltration of pro-inflammatory macrophages into a lipid-laden plaque. ITCH is an E3 ubiquitin ligase that has been shown to polarize macrophages to an anti-inflammatory phenotype. We therefore investigated the effect of ITCH deficiency on the development of atherosclerosis. ApoE−/−ITCH−/− mice fed a western diet for 12 weeks showed increased circulating M2 macrophages together with a reduction in plaque formation. Bone marrow transplantation recreated the haemopoietic phenotype of increased circulating M2 macrophages but failed to affect plaque development. Intriguingly, the loss of ITCH lead to a reduction in circulating cholesterol levels through interference with nuclear SREBP2 clearance. This resulted in increased LDL reuptake through upregulation of LDL receptor expression. Furthermore, ApoE−/−ITCH−/− mice exhibit reduced hepatic steatosis, increased mitochondrial oxidative capacity and an increased reliance on fatty acids as energy source. We found that ITCH ubiquitinates SIRT6, leading to its breakdown, and thus promoting hepatic lipid infiltration through reduced fatty acid oxidation. The E3 Ubiquitin Ligase ITCH modulates lipid metabolism impacting on atherosclerosis progression independently from effects on myeloid cells polarization through control of SIRT6 and SREBP2 ubiquitination. Thus, modulation of ITCH may provide a target for the treatment of hypercholesterolemia and hyperlipidemia.

Atherosclerosis is a vascular disease in which cholesterol accumulation within the arterial wall leads to a chronic low grade state of inflammation^1^. Several risk factors predispose to the formation of plaques including but not limited to obesity, hypertension, diabetes and hyperlipidemia^2^. The deposition of cholesterol, mainly in the form of oxidized low density lipoprotein (LDL), within the vessel wall leads to the recruitment of circulating monocytes, in an attempt to remove this excess. The activated monocytes infiltrate the plaque and attempt to clear the LDL and cell debris contained within. The excessive uptake and inability to clear these particles leads to their death with the release of further inflammatory stimuli which in turn recruit more inflammatory cells to the site of injury thus creating a perpetuating cycle^3^. Previous groups have shown that alternatively activated macrophages (M2) are able to cause plaque stabilization by the release of anti-inflammatory agents highlighting macrophage polarization as a promising target to treat or prevent the progression of atherosclerosis^4^,^5^.

ITCH is an E3 ubiquitin ligase initially discovered in the agouti locus responsible for the coat color of mice^6^. The gene encodes an 854 amino acid protein with a molecular weight of 113 Kda responsible for binding a substrate and transferring it to an ubiquitin containing E2 ubiquitin conjugating enzyme^7^. We have recently shown that the loss of ITCH shifts immune cells from a pro to an anti-inflammatory phenotype thus protecting mice from diet induced obesity complication^8^.

Here we investigate the role of ITCH deficiency on the development of atherosclerosis.

## Results

### Loss of ITCH reduces atherosclerotic burden and circulating cholesterol levels

**Figure 1:**
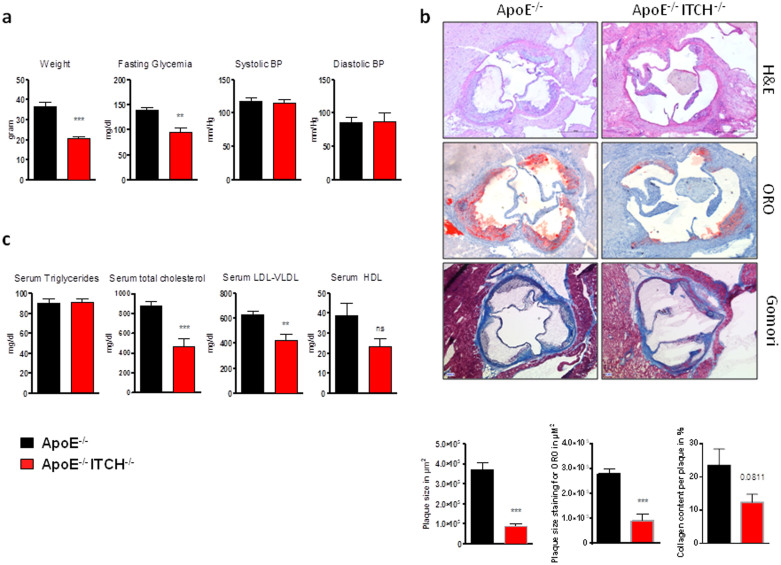
Loss of ITCH reduces atherosclerosis development, prevents weight gain, reduces glycaemia. Male 8 week old ApoE−/− and ApoE−/−ITCH−/− were fed a western diet for 12 weeks (N = 8 per group). (a) ApoE ITCH animals show reduced weight gain and improved fasting glycaemia after 12 weeks of WD but no alterations in blood pressure. (b) Representative images and measurements of aortic root plaque stained with H&E, Oil-Red-O and Gomori with mean lesion size in μm2 and %. (c) Serum analysis shows a reduction in serum cholesterol, and VLDL-LDL with no changes in serum triglycerides (N = 5 per group). Data are presented as Mean +/− SEM. ***P < 0.001 and **P < 0.01 versus ApoE^−/−^.

After 12 weeks of Western Diet (WD), ApoE−/−ITCH−/− male mice showed reduced weight gain and improved glycaemia with no differences in systolic and diastolic blood pressures (Fig. 1a) coupled to reduced plaque formation in the aortic root with a concomitant reduction in the amount of lipid and collagen within the plaque (Fig. 1b). Serum analysis of circulating cholesterol and triglyceride concentrations revealed a reduction in total cholesterol in the ApoE−/−ITCH−/− mice while there was no alteration in the serum triglyceride content between the 2 groups. The main reduction in cholesterol was at the level of LDL with the reduction of HDL not being statistically significant (Fig. 1c).

**Figure 2:**
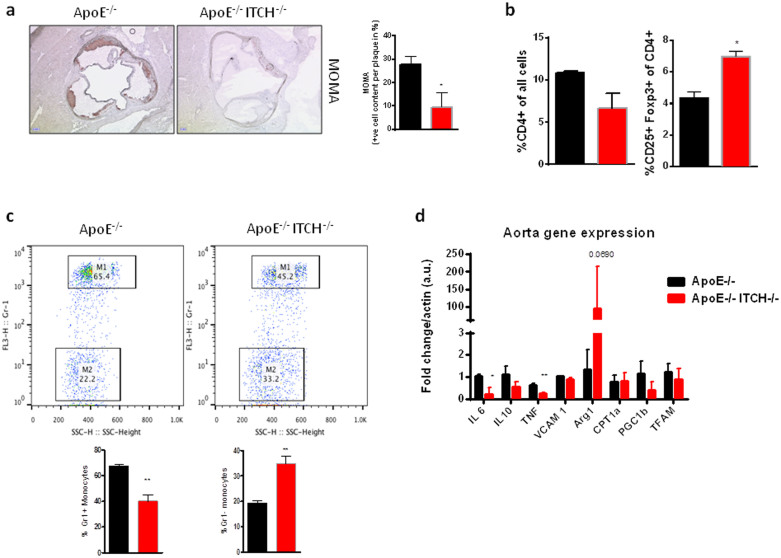
Loss of ITCH polarizes macrophages to an anti-inflammatory phenotype in ApoE−/−ITCH−/− mice. (a) MOMA-2 staining shows reduced infiltration of macrophages into the plaque. (b) and (c) FACS analysis of whole blood from ApoE−/− and ApoE−/−ITCH−/− animals shows an increase in circulating T-regulatory cells (CD4+CD25+Foxp3+) with a concomitant increase in M2 macrophages (CD115+CD11b+GR1+). (d) ApoE−/−ITCH−/− show reduced expression of pro-inflammatory markers in the aorta (N = 5 per group). Data are presented as Mean +/− SEM. **P < 0.01 and *P < 0.05 versus ApoE^−/−^.

MOMA-2 staining of the aortic root showed a reduction in macrophage infiltration in the aortic root (Fig. 2a) while FACS analysis of whole blood revealed an increase in the amount circulating T regulatory cells (Fig. 2b) and a resulting expansion of the anti-inflammatory M2 macrophages (Fig. 2c). Real Time PCR analysis of the aorta showed a reduction in inflammatory markers (Fig. 2d).

### The loss of ITCH effect is not immune cell dependent

To differentiate between the effect of ITCH loss on the immune system versus its effect on the stromal fraction we performed Bone Marrow Transplantation (BMT) experiments (Suppl. Figure 1).

**Figure 3:**
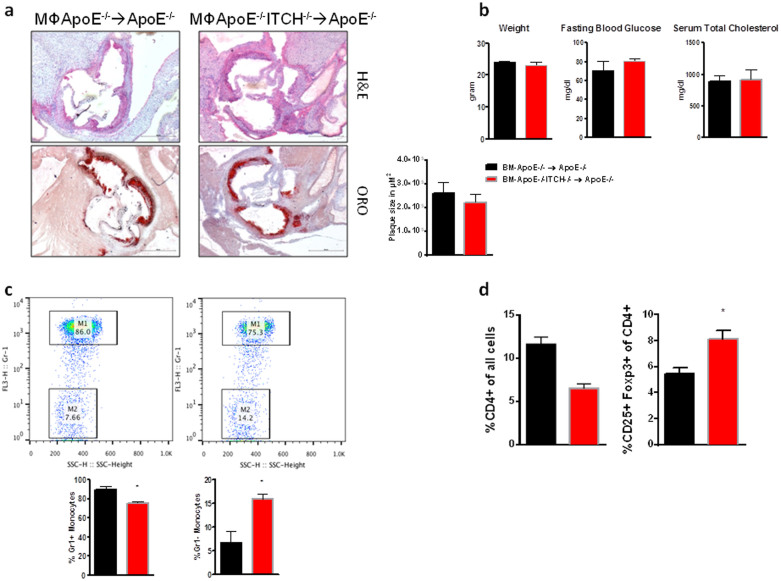
Bone marrow transplantation recreates the immune phenotype but does not affect total cholesterol and atherosclerosis development. 8 week old male ApoE mice (N = 7 per group) were bone marrow transplanted with bone marrow (BM) from ApoE or ApoE−/− ITCH−/− and then fed a western diet for 8 weeks. (a) Representative images and mean lesion size of aortic roots from ApoE−/− → ApoE−/− and ApoE−/−ITCH−/− → ApoE−/− bone marrow transplants. (b) Bone marrow transplantation does not affect weight, glycaemia or total cholesterol during 8 weeks of western diet. (c) and (d) FACS analysis of whole blood from BMT animals recreates the expansion of circulating M2 monocytes (anti-inflammatory) and T-regulatory cells as seen in the whole body knockout. Data are presented as Mean +/− SEM. *P < 0.01 versus ApoE^−/−^.

In the BMT animals we found no differences in either the development of atherosclerosis (Fig. 3a), weight, fasting glycaemia or total cholesterol (Fig. 3a). FACS analysis showed an increase in circulating FoxP3+ T- regulatory cells and the concomitant shift to an M2 polarization, similar to the whole body knockout (Fig. 3a).

### ITCH reduces hepatic lipid accumulation by increasing mitochondrial metabolism

**Figure 4:**
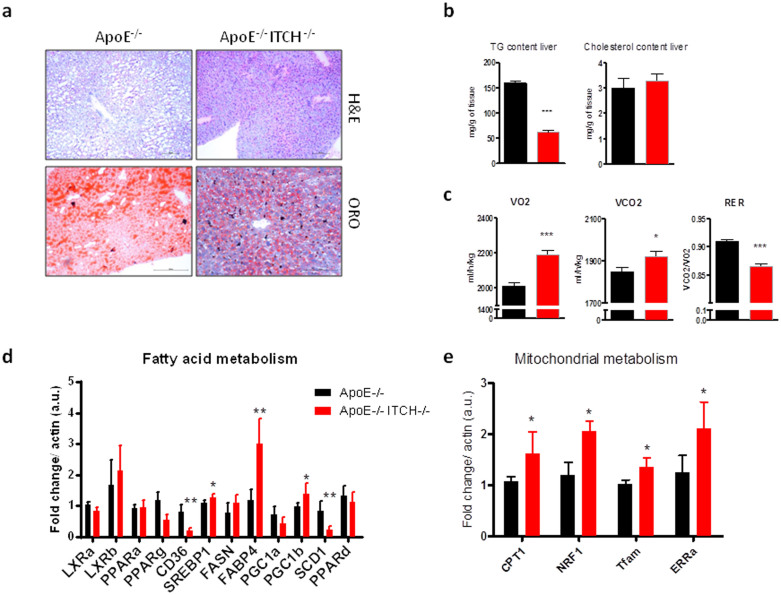
Loss of ITCH prevents fatty liver infiltration by increasing mitochondrial fatty acid oxidation. (a) Representative images of livers from ApoE−/− and ApoE−/−ITCH−/− male animals fed a western diet for 12 weeks (N = 5 per group). (b) Extraction of triglycerides and cholesterol from liver tissue shows marked reduction in triglycerides (TG) with no changes in cholesterol between the 2 groups (N = 4 per group). (c) Metabolic cage analysis of ApoE−/−ITCH−/− animals shows increased consumption of O_2_ and production of CO_2_ with a reduction in RER suggestive of increased fatty acid oxidation (N = 4 per group). (d) analysis of fatty acid metabolism genes in the liver (N = 5 per group). (e) Increased gene expression of genes involved in mitochondrial metabolism in the liver (N = 5 per group). Data are presented as Mean +/− SEM. ***P < 0.001, **P < 0.01 and *P < 0.05 versus ApoE−/−.

As the liver is the main site of triglyceride and cholesterol synthesis we next examined the hepatic tissue. Histological analysis showed conserved liver architecture with reduced accumulation of neutral lipids in ApoE−/−ITCH−/− animals (Fig. 4a). Interestingly, opposite to the serum concentrations we found a reduction in triglyceride content in the liver tissue, while the cholesterol content was comparable between the 2 groups (Fig. 4a).

To determine the underlying mechanism behind the reduced triglyceride accumulation in the liver we performed indirect calorimetry measurements. These revealed an increased production of VO2 and VCO2 coupled with a mild but significant decrease in the Respiratory Exchange Ratio (RER). Together these values suggest that ApoE−/−ITCH−/− have a higher metabolic rate couple to an increased reliance on fatty acids as an energy source (Fig. 4c and Suppl. Fig. 2c). A limited gene transcription analysis of the liver revealed a reduction of CD36 and SCD1 and increased transcription of FABP4, SREBP1 and PGC1beta (Fig. 4d). Since there was no increase in the classical activators of fatty acid oxidation such as PPARalpha we then investigated markers of mitochondrial oxidation including CPT1, NRF1, TFAM and ERRalpha which we found to be significantly upregulated in the ApoE−/−ITCH−/− animals (Fig. 4e).

### Loss of ITCH reduces ubiquitination of SIRT6

**Figure 5:**
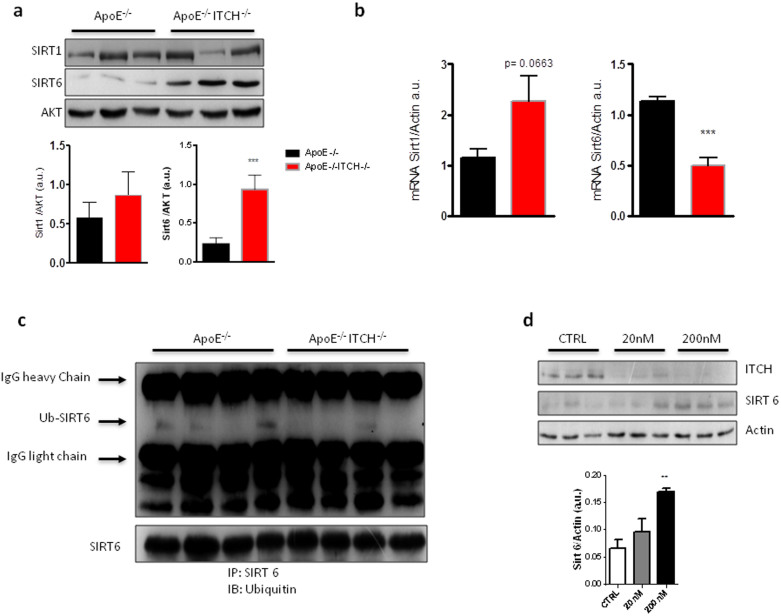
ITCH modulates SIRT6 to influence liver triglyceride metabolism. (a) and (b) Loss of ITCH leads to selective upregulation of SIRT6 protein levels in the liver despite a reduction in mRNA expression suggesting post translational modification (N = 4). (c) Representative images of immunoprecipitation of liver SIRT6 and immunoblotting with anti-Ubiquitin antibody showing decreased ubiquitination of SIRT6 in the ApoE−/−ITCH−/− (N = 4). (d) Knockdown of ITCH by increasing concentrations of SiRNA in HepG2 cells increases the protein levels of SIRT6 (AKT = Protein kinase B). Data are presented as Mean +/− SEM. ***P < 0.001 and **P < 0.01 versus ApoE^−/−^.

The metabolic phenotype of reduced weight gain, improved glycaemia, reduced liver triglycerides and increased mitochondrial oxidation strongly recalls the phenotype of Sirt6 transgenic mice. Indeed, western blotting revealed an upregulation of SIRT6 in the liver while there were no changes in SIRT1 (Fig. 5a). Interestingly, while protein levels of SIRT6 were increased, its transcription level was downregulated in the ApoE−/−ITCH−/− (Fig. 5b) suggesting that the loss of ITCH from the liver increases the expression of SIRT6 possibly by reducing its ubiquitination. Consequently, when we immunoprecipitated SIRT6 from the liver and immunoblotted with ubiquitin we found that in the livers of ApoE−/−ITCH−/−, SIRT6 was less ubiquitinated (Fig. 5c).

SiRNA knockdown of ITCH in HepG2 cells confirmed the link by increasing SIRT6 in a dose dependent manner (Fig. 5d).

### Loss of ITCH affects cholesterol metabolism through SREBP2

**Figure 6:**
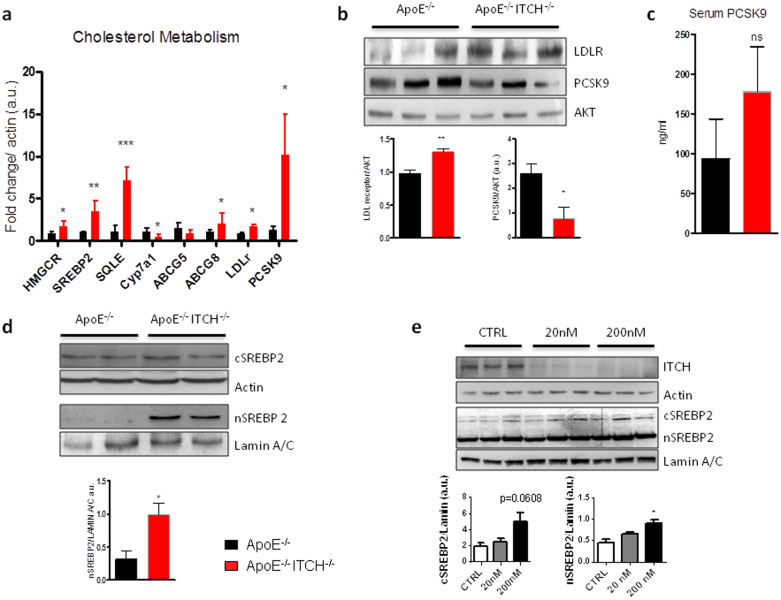
ITCH modulates cholesterol levels through reduced degradation of nuclear SREBP2. (a) mRNA expression analysis for genes involved in cholesterol metabolism in the liver (N = 4). (b) Representative western blots for LDL receptor and PCSK9 from livers of ApoE−/− and ApoE−/−ITCH−/− (N = 6 per group). (c) PCSK9 serum levels from ApoE-/- and ApoE-/-ITCH-/- mice (N = 6 per group) (d) Representative western blot and densitometry of nuclear extracts from livers of ApoE−/− and ApoE−/−ITCH−/− mice. (e) Effect of ITCH knockdown by increasing concentrations of SiRNA in HepG2 cells on ITCH and SREBP2 (cytisolic, c; nuclear, n) protein levels. Data are presented as Mean +/− SEM. ***P < 0.001, **P < 0.01 and *P < 0.05 versus ApoE^−/−^.

To investigate in more depth the reduction of circulating cholesterol we measured the expression of several genes involved in the synthesis, excretion and re-uptake of cholesterol. Unexpectedly, in the ApoE−/−ITCH−/−, we observed an upregulation of several genes involved in cholesterol synthesis, while genes involved in cholesterol excretion were minimally modulated (Fig. 6a).

With a gene expression profile indicative of increased cholesterol production we next analyzed cholesterol re-uptake and found that the LDL-receptor is upregulated at a transcriptional as well as protein content level (Fig. 6a,b). However, concomitantly we found increased expression of PCSK9 which is progressively more secreted thus accounting for the reduced protein content in the liver (Fig. 6a–c). Most of the genes of cholesterol synthesis are directly or indirectly regulated by SREBP2 and we therefore next measured the nuclear content of the active form of SREBP2, which we found to be increased in the ApoE−/−ITCH−/− (Figure 6d). In our BMT animals ITCH expression in the liver was not altered and we consequently did not observe any effects on the expression of the LDL receptor (Suppl. Fig. 3).

ITCH SiRNA knockdown in HepG2 cells resulted in increased nuclear localization of SREBP2 (Fig. 6e).

## Discussion

We herein show for the first time that the loss of ITCH results in a reduced development of atherosclerosis coupled with reduced hepatic fatty infiltration in the hypercholesterolemic ApoE−/− mouse model through reduced clearing of SREBP2 and SIRT6 respectively.

While the loss of ITCH also results in a shift towards an anti-inflammatory M2 phenotype, this appears to be uncoupled, in our model, from the development of atherosclerosis, suggesting that the main effect is driven by the reduction in the concentrations of circulating lipoproteins. Indeed this effect is lost when performing bone marrow transplantation. It has recently been suggested by Ferrante et al. that, in the adipose tissue, it may not be the shift towards the classical M1 macrophage that is responsible for the deleterious effects of increased cytokine production, but rather the overall expansion of the monocyte population conferring insulin resistance^9^. Our results further add to this theory by showing that despite the increase in M2 macrophages, the effect on atherosclerosis appears mainly mediated by the effect on the stromal tissues and the ensuing reduction in cholesterol.

We therefore postulate that the loss of ITCH in the liver hampers the breakdown of SREBP2 thus leading to an increased expression of the LDLr, which is enough to compensate for the increased cholesterol synthesis and levels of circulating PCSK9. As a matter of fact the BMT animals showed increased levels of cholesterol as the bone marrow transplantation did not effectively reduce liver levels of ITCH resulting in a selective upregulation of PCSK9, a mechanism that remains to be explored.

The main regulation of SREBP2 is through the intracellular content of cholesterol. Increased intracellular cholesterol usually inhibits the cleavage of the inactive form of SREBP2 to its active, nuclear form. The active nuclear form of SREBP2 is mainly regulated by ubiquitination dependent degradation^10^ but is also able to induce its own transcription^11^. Considering that there are no differences in the cholesterol content of the liver, the stimulus for SREBP2 to be translocated ought to be the same.

The clinical importance of SREBP2 has been clearly demonstrated in patients with hypercholesterolemia in whom the treatment with a statin results in the upregulation of the LDL receptor through increased activation of SREBP2. While there has been some discussion regarding the concomitant upregulation of PCSK9 there are currently promising agents under development, which specifically target PCSK9 thus allowing, further decreases in LDL levels.

Furthermore, our data clearly show that in a model of hepatic lipid overload^12^, the modulation of ITCH is able to reduce the accumulation of triglycerides into the liver. Taken together with the increased reliance on fatty acids as a main energy source, as suggested by the increased V0_2_ consumption and CO2 production shown in our calorimetric studies and the upregulation of several mitochondrial markers of oxidative metabolism in the liver of ApoE−/−ITCH−/− mice, ITCH emerges as a direct regulator of lipid metabolism in the liver. Since the bone marrow transplant experiments de-emphasized the metabolic role of a Th2 bias, at least in this model, we consequently looked for targets at a hepatocyte level. Despite several putative targets, such as the P73/PML/PPARδ pathway (Suppl. Fig. 4) that we found unaffected in our model but are relevant in other contexts^13^,^14^, our data revealed SIRT6 as a new target for ITCHE3 ubiquitin ligase that recapitulate our results on the triglyceride metabolism.

Despite SIRT6 having been shown to be able to downregulate SREBP2 to reduce cholesterol production^11^, our data show this effect to be lost in ApoE−/−ITCH−/− mice. One potential explanation for this discrepancy may be the use of a western type diet, since it has been shown that a diet enriched in lipids increases the expression of hepatic SREBP2^15^, potentially bypassing the repressive effect of SIRT6. In addition, insulin sensitivity may also contribute to this phenomenon, given that SREBP2 expression is downstream from insulin receptor signalling and the deficiency of ITCH increases hepatic insulin sensitivity under a diet enriched in lipids^8^,^16^,^17^,^18^. Interestingly the effect of ITCH on cholesterol metabolism is influenced by combined genetics/nutritional stimuli for atherosclerosis, since in ITCH−/− mice fed a high fat diet we found improved tryglyceride metabolism^8^, but no differences in the expression of genes involved in cholesterol metabolism (Suppl. Fig. 5).

In summary we show that the loss of ITCH reduces atherosclerotic development by preventing the clearance of SREBP2 and thus upregulating the LDL receptor mediated reuptake of LDL into the liver. Further to this beneficial effect, we show that the loss of ITCH from the liver increases the expression of SIRT 6 by reducing its ubiquitination thus protecting from fatty infiltration. In summary we suggest that the modulation of ITCH at hepatic level is an interesting target to be further exploited for effects on hypercholesterolemia and hepatic steatosis.

## Methods

### Mouse model and metabolic studies

All animal studies were approved and carried out in strict compliance with the University of Tor Vergata Institutional Animal Care and Use Committee (IACUC) guidelines and in accordance with the “Guide for the Care and Use of Laboratory Animals” (1996) by the Institute of Laboratory Animal Research Commission on Life Sciences (ILARCLS, National Research Council, Washington, D.C.).

Itch−/− mice were crossbred with ApoE−/− mice to generate heterozygotes. These were then interbred to generate a pure lineage, which was then backcrossed into ApoE−/− for 5 generations. ApoE−/−Itch−/− mice and ApoE−/− littermates were maintained on 12 hrs light and dark cycles under controlled environmental conditions, with free access to water and food. Studies were performed only in male mice. Where described, after weaning, animals were fed a western diet (45% calories from fat and 1,5% cholesterol) for 8 or 12 weeks. Metabolic testing procedures and ITCH−/− fed high fat diet (HFD) were previously described^8^. Tissues were collected after overnight fasting snap frozen in liquid nitrogen and stored at −80C.

Blood was collected after overnight fast, allowed to clot at room temperature and then centrifuged at 2000 G for 20 mins at 4C to obtain serum. Total cholesterol, HDL and the VLDL/LDL fraction were measured by colorimetric assay according to the manufacturers instructions (Abcam).

To determine atherosclerosis in the outflow tract and valve area the top half of the heart was embedded in Optimized Tissue Compound (Tissue Tek) and stored at −80 until sectioning. Serial 8 micrometer sections where all 3 valve leaflets were visible were stained with Oil Red O and mounted with gelatin.

### Cholesterol and triglycerides assay

Cholesterol and triglycerides were extracted from liver tissues and analyzed using the Total cholesterol Assay Kit and Triglyceride Quantification Kit (Cell biolabs, inc.) according to the manufacturer's instruction.

### Bone marrow transplantation

Bone marrow transplantation (BMT) studies were performed as previously described^8^. In short, 6–8 week old WT recipient mice were given busulfan (Sigma_Aldrich) (20 mg/kg/day) for 4 days (days −7, −6, −5 and −4) followed by cyclophosphamide (Sigma_Aldrich) (100 mg/kg/day) for two days (days −3 and day −2). Mice were rested on day −1, and BMT was performed on day 0. On day 0 bone marrow cells were harvested from ApoE−/− and ApoE−/−Itch−/− mice femurs and tibias by flushing with PBS. After washing and counting 5 × 10^6^ cells bone marrow cells were then injected in the retro-orbital venous plexus of the recipient ApoE−/− mice. Feeding with western diet started 3 weeks after engraftment. PCR for the Itch−/− genotype was performed on DNA extracted from blood of recipient mice 8 weeks after BMT to confirm the efficiency of this BMT technique.

### Flow cytometry

FACS analysis was performed as previously described^8^. In brief, 100 microliters of blood were collected retroorbitally and anticoagulated with Heparin. Red blood cells were lysed with RBC Lysis Buffer (EBioscience) and then stained with CD115-APC (Milteny Biotec), CD11b-FITC (Miltenyi Biotec) and GR1-PerCP (Miltenyi Biotec). Monocytes were defined as CD115+ and CD11b+, while Gr1 was used to mark pro-inflammatory monocytes.

For labeling of T-regulatory cells we used a staining kit according to the manufacturers instructions (Milteny Biotec). T-Regs were defined as CD4+, CD25+ and FoxP3+.

Samples were analyzed using a FACScalibur (BD bioscience) running BD Cellquest Pro and analyzed with Flow JO (TreeStar Inc., Ashland OR).

### Cell culture experiments

HepG2, human hepatoma cells (ATCC, Rockville, MD), were maintained in RPMI containing 10% fetal bovine serum (FBS), 100 U/ml penicillin, and 100 mg/ml streptomycin (Invitrogen, Life Technologies). Cells were transfected with *Itch* siRNA using the Nucleofecteor AMAXA transfection kit according to the manufacturer's instructions with the use of a Nucleofector machine (Amaxa/Lonza). In short, cells 1 × 10^6^ were resuspended in 100 μL of KIT V nucleofection solution (Amaxa/Lonza) with stated concentrations of *Itch* siRNA or control scramble sequences (Ambion, Texas), permeabilized and then plated and incubated at 37°C for 2 days before harvesting for Protein and RNA studies.

### Nuclear extracts

Nuclear protein was extracted by using the NE-PER kit according to the manufacturers instructions (Thermo scientific).

### Western blot and immunoprecipitation

Western blotting and immunoprecipitation were performed with the following antibodies: SIRT1 (ab28170), SIRT6 (ab62739), SREBP2 (ab30682), LDL receptor (ab52818), ITCH (BD pharmigen 611198), PCSK9 (R&D biosystems AF3985). Actin and Lamin A/C (Santa Cruz Biotechnology) and AKT (CST 9272) were used for control.

Immunoprecipitation with SIRT6 was performed as previously described^18^. Immunoprecipitate was separated on SDS, transferred to Nitrocellulose and probed with anti-Ubiquitin (Cell signaling technology).

### Statistical Analysis

Results of the experimental studies are mean ± SD or SEM as specified. Statistical analyses were performed using the one-way ANOVA or unpaired Student's *t* test with GraphPAD Prism 5.1. Values of *p* < 0.05 were considered statistically significant.

## Supplementary Material

Supplementary Information Supplementary Figures
