# Supplementary material for: ITCH modulates SIRT6 and SREBP2 to influence lipid metabolism and atherosclerosis in ApoE null mice
Source: Sci Rep. 2015 Mar 17;5:9023. doi: 10.1038/srep09023 (PMC4361881; doi:10.1038/srep09023)
Supplement: Supplementary Information — Supplementary Figures [file srep09023-s1.docx]

**ITCH modulates SIRT6 and SREBP2 to influence lipid metabolism and atherosclerosis in ApoE null mice**

R. Stöhr^1^; M. Mavilio^1^; A. Marino^1^; V Casagrande^1^; B. Kappel^1^; J. Möllmann^3^; R. Menghini^1^; G. Melino^2,4^; M. Federici,^1,5^.

1. Department of Systems Medicine University of Rome “Tor Vergata”
2. Department of Experimental Medicine and Surgery University of Rome “Tor Vergata”
3. Medizinische Klinik I, University Hospital Aachen
4. Medical Research Council, Toxicology Unit, Leicester LE1 9HN UK
5. Center for Atherosclerosis, University Hospital “Policlinico Tor Vergata”, Rome

*Address correspondence to: Massimo Federici, Department of Systems Medicine University of Rome “Tor Vergata” Via Montpellier 1, 00133 Rome Italy

Phone: +39-06-72596889 Fax: +39-06-72596890 E-mail: federicm@uniroma2.it

SUPPLEMENTARY FIGURES


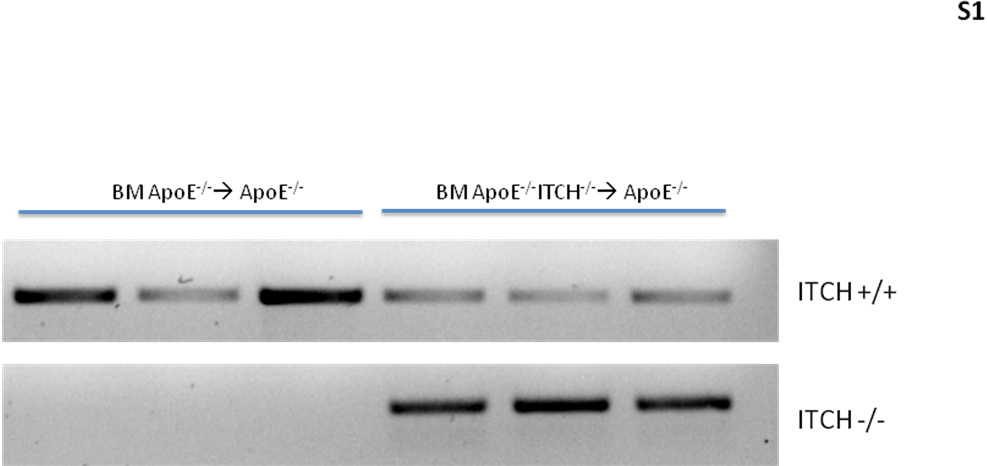


**Supplementary Figure 1** PCR analysis of Bone marrow transplantation experiments shows chimerism of the transplanted animals.


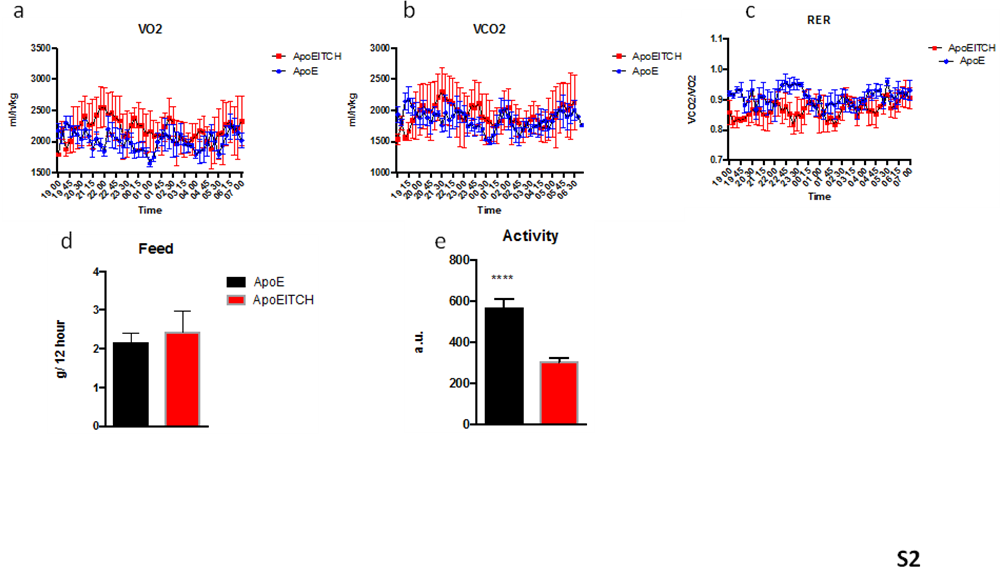


**Supplementary Figure 2**  Loss of ITCH increases reliance on fatty acids as an energy source. ApoE-/- ITCH-/- animals show increased levels of O2 consumption (A) and CO2 (B) production with a lowered Respiratory Exchange Ration (RER) (C). No changes were seen in food consumption (D) while activity was reduced in the ApoE-/- ITCH (-/-) animals (N=4 per group)


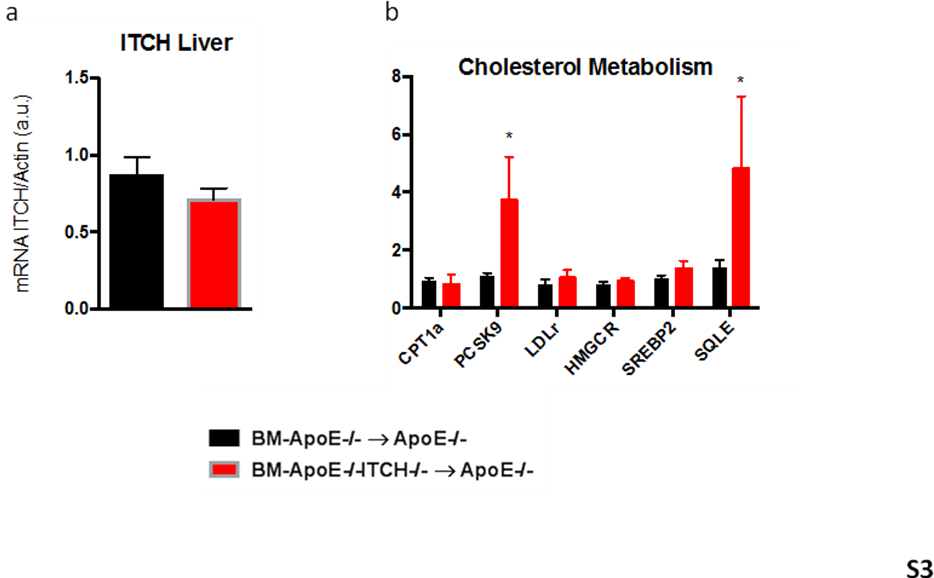


**Supplementary Figure 3** Bone marrow transplantation does not affect ITCH liver levels.

Bone marrow transplantation does not result in a downregulation of ITCH in the liver (A) and consequently does not affect the cholesterol metabolism in the same manner as the total body knockout (B) (N=4 per group)


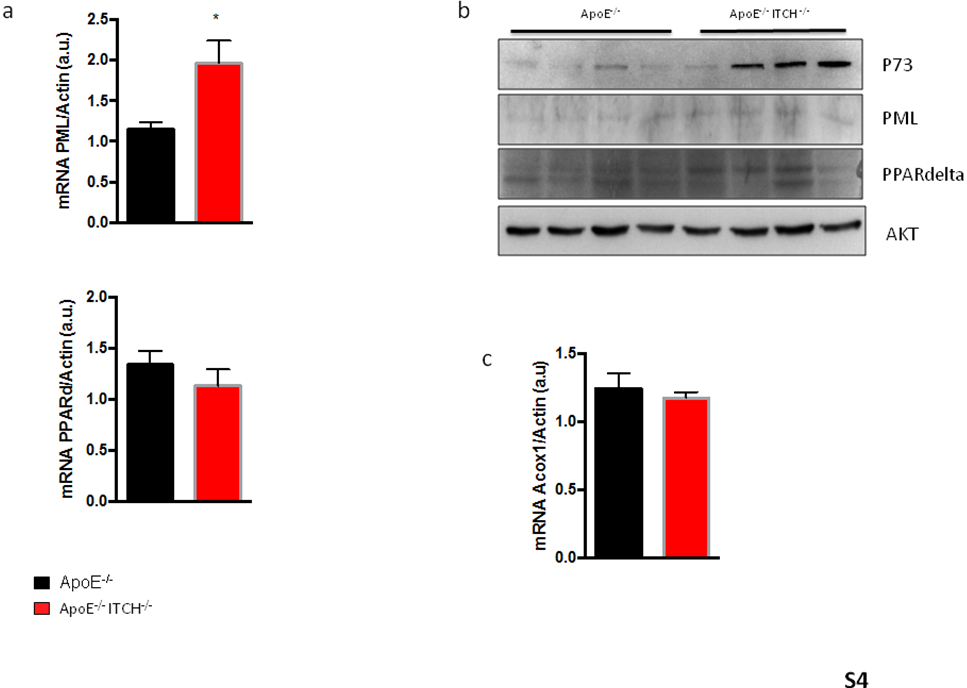


**Supplementary Figure 4: A)** RT-PCR and B) western blots of liver tissue show that the P73-PML-PPARδ is not affected in the livers of ApoE -/- ITCH-/- mice. While there is the expected increase in the protein content of P73 in the livers of ApoE-/- ITCH-/- there is no upregulation of the protein content of PML and PPARδ. C) Furthermore, the direct target of PPARδ, Acox1 is unaffected in the livers of ApoE -/- ITCH -/- mice.


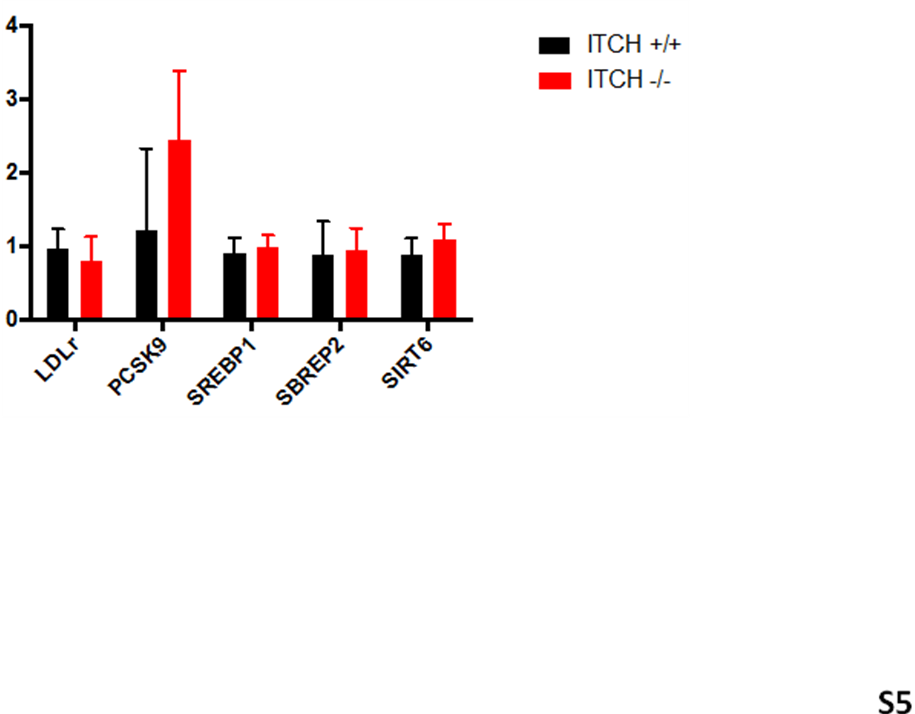


**Supplementary Figure 5:** RT-PCR of liver tissue from ITCH-/- mice does not show any alterations in the expression of genes related to cholesterol metabolism. .
